# Treatment with Venetoclax for Chronic Lymphocytic Leukemia with the Highest Known White Blood Cell Count: Safe and Effective

**DOI:** 10.4274/tjh.galenos.2021.2021.0435

**Published:** 2021-12-07

**Authors:** Mehmet Sönmez, Merve Kestane, Osman Akıdan, Nergiz Erkut, Özlen Bektaş

**Affiliations:** 1 Karadeniz Technical University Faculty of Medicine, Department of Hematology, Trabzon, Turkey

**Keywords:** Chronic lymphocytic leukemia, Venetoclax, Highest white blood cell count

## To the Editor,

Venetoclax is a potent, selective inhibitor of B-cell lymphoma 2 (BCL-2) that is a key regulator of apoptosis and is used for the management of chronic lymphocytic leukemia (CLL) either alone or in combination. Inhibition of BCL-2 induces apoptosis, leading to rapid tumor debulking and high response rates in cases of CLL. The toxicity profile of venetoclax includes manageable hematologic toxicities such as neutropenia, gastrointestinal adverse events, and tumor lysis syndrome (TLS). The risk of TLS can be reduced by a slow dose ramp-up, strict monitoring, and adequate prophylaxis [1,2,3].

**Figure 1:**
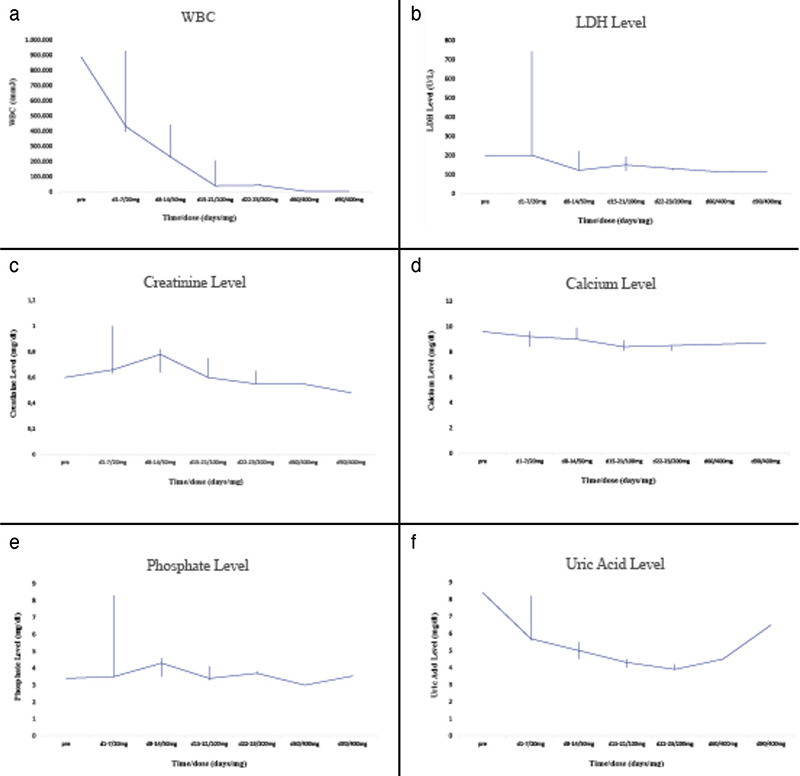
Trends of tumor lysis syndrome parameters during the first 3 months of treatment. Changes of (a) white blood cell (WBC), (b) lactate dehydrogenase (LDH), (c) creatinine, (d) calcium, (e) phosphate, and (f) uric acid levels.

An 86-year-old female patient presented to the hospital with fatigue, weight loss, night sweats, shortness of breath, and weakness. She had been diagnosed with CLL 10 years previously and she subsequently received four lines of chemoimmunotherapy. During the COVID-19 pandemic she did not visit the hospital for CLL follow-up. Her white blood cell count was 925,190/µL with 90% lymphocytes. Hemoglobin level was 5.2 g/dL and platelet count was 128,000/µL. Lymphocytes expressed CD5, CD19, CD23, CD200, and CD20. Other laboratory investigations including urea, electrolytes, and liver function tests were all within normal limits. In computed tomography scans of the neck, thorax, abdomen, and pelvis, she was observed to have splenomegaly (150 mm) and paraaortic, supraclavicular, axillar, and mediastinal multiple, differently sized (32x21 to 22x13 mm) lymphadenopathies. Venetoclax treatment was planned with a 5-week dosing ramp-up (20 mg, 50 mg, 100 mg, 200 mg, 400 mg) with adequate tumor lysis prophylaxis (allopurinol and 1.5-2 L of fluid daily) and close monitoring. In the third week, COVID-19 infection was detected. The venetoclax treatment was continued without any change and the patient was discharged with no complications. Data obtained from laboratory monitoring are presented in Figure 1.

We observed rapid tumor debulking without complications and the disappearance of symptoms probably caused by venetoclax-induced apoptosis. We conclude that venetoclax is an effective and safe treatment option for CLL [4,5,6].
